# Motivating medical students for social accountability in medical schools

**DOI:** 10.30476/jamp.2020.84117.1128

**Published:** 2020-04

**Authors:** MAHBOOBEH MOHAMMADI, MEHDI BAGHERI, PARIVASH JAFARI, LEILA BAZRAFKAN

**Affiliations:** 1 Islamic Azad University, Bandar Abbas Branch, Iran; 2 Department of Educational Management, Islamic Azad University, Bandar Abbas Branch, Iran; 3 Department of Educational Management, Islamic Azad University, Science and Research Branch, Tehran, Iran; 4 Clinical Education Research Canter, Shiraz University of Medical Sciences, Shiraz, Iran

**Keywords:** Motivation, Medical student, Accountability, Qualitative Research, Content analysis

## Abstract

**Introduction:**

As health professionals, physicians are accountable for their professional practice.  The aim of this study was to explain the medical students’ motivation to attain social accountability in medical schools, based on the experience of both students and faculties.

**Methods:**

We conducted a qualitative conventional content analysis research in Shiraz University of medical sciences in Iran since 2018 through purposive, snowball sampling. The data were collected through semi-structured interviews with 35 participants i.e., medical students and teachers. Coding was carried out by conventional content analysis.

**Results:**

We drew four themes and ten related subthemes and the central variable explains the motivation of medical students toward social accountability and makes a link among the subthemes, was purposeful beliefs and behavior. The key dimensions during motivational process which generated the social accountability development in medical students consisted of social culture of medicine, medical school reality, teaching and learning strategy and creating purposeful beliefs and behavior. Also, eight subthemes of individual motivation, content motivation process motivation, attending to the outcomes of the curriculum, traditional routine centered curriculum, observational learning, role modeling, hidden curriculum, respect for social values and norms and benefitting the society emerged which explain the process of motivate for social accountability by creating purposeful beliefs and behavior in medical students.

**Conclusions:**

The core variable of motivation toward social accountability must be reflected in future developmental programs, curriculum planning and training general physicians. In other words, the best efforts for purposeful beliefs and behavior in medical students, must be made to improve motivation toward social accountability.

## Introduction

Medical schools are committed for training competent students who are committed to the health of the community and play the roles of delivering health as well as preventive, educational, managerial and treatment services in individual, family, and community levels. Accountability is a requirement of an active and key physician in the workplace and social accountability is among the important concepts in medical schools’ objectives and curricula ( 1 - 3 ).

 WHO defines social accountability as “commitment to performing educational and research activities and services in the same line with the region, society, and the country where they intend to deliver services” ( 4 ). The important point is the existence of adequate motivation which is one of the significant factors in learning and can affect the students’ behavior to be shaped; also, the educational motivation is considered as an impetus for the students’ activities. Thus, any attempt for the improvement of this impetus in the learners can be of utmost importance ( 5 , 6 ). 

The medical students’ high motivation is important not only for the medical community, but also for the future of our country’s health system ( 7 ). Recognition of the ways to boost the students’ motivation, the factors affecting it, and awareness of the students’ individual differences, especially in a responsive system, can contribute to compiling regulations, providing facilities, and planning successfully for the physicians’ future career, improvement of the educational issues, educational guidance, and provision of health services in line with the society’s needs for future. In this regard, some revisions have been made in educational curricula and learning strategies ( 8 - 10 ).

Several studies have been conducted on this subject. Kwizera & Iputo have necessitated a holistic view to medical education presented to South African students and considered it as a form of social accountability in medical schools. They believe that social accountability in medicine is autheticized only when it is included in the basic philosophy of medical schools. They conclude that unwritten rules of ubuntu who had been the governor of South Africa for many years are potentially able to play a role as the medical schools’ core principles in their country, which lead to the expansion of social approaches to physician’s accountability ( 11 ). In another study, Gibbs & McLean have necessitated preparation of a global accountability model in international medical education systems. They believe that graduates of medicine should acquire necessary skills in medical schools to be responsive to extensive global communities. They also assert that such a model should comprise of all the responsibilities pertinent to all aspects of their professional development, so that it can be used in more communities than the one it has been developed in ( 12 ). 

Therefore, given the importance of qualitative studies and their influential role in clarifying the ambiguous and unknown areas, grounded theory is suggested to be adopted since this method is efficient in answering questions about human’s internal conceptions and interpretations; in other words, this is the best approach to describe life experiences and their inherent social processes. In this study, we aimed to shed light on the motivation processes in students and determine how various factors and indicators increase the students’ motivation, leading to their social accountability. We introduced a model for the creation of motivation, using the effect of these factors on the end result and the role of students, teachers and educational environment in this process. Therefore, our main objective of this study was making a model of creating motivation for social accountability in medical students in Shiraz University of Medical Sciences, using grounded theory.

In this study, we discovered the themes that clarify How Medical students are motivated for social accountability in medical schools in Iran, and developed a conceptual framework/model to explain this motivation for accountability based on the experience of both medical students and their teachers.

## Methods

This study was conducted in Shiraz University of medical sciences. This study is part of a qualitative study based on an Inductive content analysis in an organized form ( 13 - 15 ). The investigator’s tenacity in using conventional content analysis was to define and clarify a phenomenon in the social condition and to identify the essential processes working within ( 16 ).

In this study, 35 subjects participated in the study comprising of 15 faculty members of medical schools and 20 undergraduate medical students selected by purposive, snowball sampling with maximum variation; we continued the sampling procedure to data saturation. The basis for theoretical sampling was the queries that emerged during data analysis ( 17 ). We selected the participants from varied levels of teaching, different work experiences, In addition, we interviewed ordinary and talent students, who have been active and responsible in teaching and learning. The inclusion criterion was 3 years of work experience in teaching, and the exclusion criterion was the unwillingness to participate in the study. Firstly, we collected data in Shiraz University with the help of an educational administrator Students were selected based on their enthusiasm to participate. Data saturation was attained when no new records emerged in the last four interviews. 

Data were collected through semi-structured interviews with the participants selected from December 2018 to September 2019. The interviews began with the following questions: “Can you talk a bit about a typical day at your medical training?”; “How do you define accountability in medicine?”; “What circumstances inﬂuence the medical students, motivation during basic and clinical education?”, and “How are medical students motivated to be accountable in medical education?” Based on the participants’ responses, the questions progressively became more focused. Each interview lasted from 30 to 90 min. All the interviews were audio-taped and transcribed for analysis. At the end of each session, the participants were given an opportunity to raise other important topics not mentioned during the interview, followed by data collection and analysis which are simultaneously performed based on the principles of grounded theory; analytic thoughts and queries that arose from one interview session were carried on to the next one ( 14 - 18 ).

We performed data collection and analysis by using a conventional content analysis method. We extracted the basic concepts or meaning units from the gathered information. Then, more general concepts were formed by grouping similar concepts into one theme. The themes emerged during the interviews. Then, the constructs were compared to form tentative categories. To check the relevance of data to categories, the researcher asks queries related to certain classifications and returns to the data to seek evidence.

In terms of trustworthiness, we used the Lincoln and Guba’s criteria including credibility, dependability, conformability and transferability ( 19 , 21 ).

To increase credibility, we collected data from students and teachers in different situations and wards in Shiraz University of medical sciences, and the credibility of data was confirmed by five specialists in qualitative research. Also, some participants meticulously rechecked the researchers’ description and interpretation of their experiences. Long engagement of the researcher in field helped the data credibility. In this way, the process of data collection and analysis lasted for 12 months. The use of the maximum variation sampling of contributors added to the dependability and conformability of data. As a final point, to accomplish transferability, we meticulously described the data in this article, so that readers can judge transferability of data themselves.

### *Ethical considerations*

This study was approved by the Ethics Committee of Islamic Azad University of Hormozgan (5965). The participants were informed about the research aim and interviews. Informed consent was obtained for conducting and recording the interview. The confidentiality of the participants’ information was maintained throughout the study.

## Results

In this study, the mean age of the faculty members and students was 46.25 ± 11.30 and 23.34 ± 4.64 years, separately. All the faculty members and only two students were married. Four themes and ten interrelated sub-themes emerged from the data (Table 1). The main variable, which explains the process of motivating the students for social accountability as the phenomenon of concentration and which makes an association among the categories, was purposeful beliefs and behavior. 

**Table1:** How medical students are motivated for social accountability: themes, subthemes and meaning unit

Theme	Subthemes	Meaning unit
Social culture of medicine	Individual motivation	Talent, personal interest, self-awareness, meeting students' needs,
	Content motivation	attractiveness of medical topics
		Being scientific
		The same content in total world
	Process motivation	effective job
		Helps all the people respect this profession
		Value the medical field.
		Dignity of the field to the community
		Enjoy being a doctor.
Medical school reality	Pay attention to the outcomes in the curriculum	Outcome--based curriculum
		Attention to the competence
		Attention to the outcomes
		capability
		Effective evaluation
	Traditional routine-centered curriculum	Traditional routine rule
		Traditional lecture
		Traditional classroom
Teaching and learning strategy	Observational learning	Peer assisted learning
		Self-directed learning, participatory teaching learning strategies
	Role modeling,	teacher as the role model
		residents as the role model
		Peer as the role model
	Hidden curriculum	Learning through a hidden curriculum
purposeful beliefs and behavior	Respect for social values and norms	Belief in medicine
		continuity of accountability Respect for social values and norms, social justice
		purposeful behavior
	Attention to the benefits of patients and society clearly	Patient care must be clear Patient care must be documented community is sensitive to patient rights

### *Theme 1: Social culture of medicine*

In this theme, based on the experience of participants, a student who attends university is affected by the culture and hegemony of the medical community, and will be directed to motivate for obtain competence for social accountability from the outset. In this category, there are the three subgroups of content motivation, process motivation.

### *Individual motivation*

Participants, personal experiences, cite individual personality talent, ethical commitment, professional ethics, and self-awareness are the leading factors in the field of medicine and accountability to the community. Based on the experiences of the participants, usually the most talented students are attracted to the medical field, and many of the experiences that they quote indicate self-awareness in course selection. Self-awareness implies that a person is aware of feelings and behaviors of their own and also aware of their strengths and weaknesses. They do not see their successes and failures as accidental and contemplate on the causes for each incident. Being self-aware means that one holds himself accountable for his choices in the future. Self-awareness in the field of recognizing situations, communicating with others, and being conscious has a significant impact on the motivation and empowerment process. One of the students reported:

*"... What I want and what I like to be in the future depends a lot on how much I know myself. If I'm not capable of understanding high level content, I'm a comfortable person, if I'm not dutiful, If I don't like other people, how can I be accountable to them? This self-awareness is a key in any field...”* (Participant No. 15).

### *Content motivation*

The specialties in medical work, the realism and the context provider of theoretical and practical education create content motivation in terms of the attractiveness of medical topics which attracts students, which leads students to try for learning science and act to be more accountable in the community. One of the students said:

*"Medical work is in fact a specialty. Because not everyone can do it, it is automatically interesting. And the other thing is that the medical stuff itself is interesting enough and make motivation to learn ... which science is sweeter and more diverse like medical knowledge? "* (Participant No. 14).

### *Process motivation*

Concerning process motivation, participants also believe that the attractiveness and dignity of the field to the community and people around the world are among the factors that make the medical field attractive to entrance exam candidates who compete for it. People and families have respected this profession and its owners from past. One professor said:

*"The community does not know some facts about medicine right now, but the respect that people have for this field and the doctors and the trust that they have is considerable; we have to be grateful and prepare ourselves for good service."* (Participant No. 24).

### *Theme 2: Medical school reality*

The reality of medical school education is a theme that the participants agree on. Most participants have an emphasis to express their experiences on the necessity to pay attention to community needs and learners and their impact on educational outcomes and student evaluation, responsibility and accountability. They believe that if the needs of the community and the students are provisioned in the curriculum at the beginning, the outcome-based is designed, and students are evaluated for the same purpose, and we can expect to have the best accountability in the future from student. Otherwise, if we follow routine and traditional programs and make no changes to the curriculum, students will have no incentive to respond in the future.

### *Paying attention to the outcomes in the curriculum*

One of the problems with this, according to the participants, is that sometimes in designing and teaching different courses, educational outcomes are not given much attention. In cases where the outcomes are designed according to need, students are most motivated. One of the students said:

*"... An important problem for us is that why these lessons will be taught ultimately, and what is the purpose of teaching these topics in the end? When tomorrow I forget everything this work is all in vain…”* (Participant No. 18).

### *Traditional Routine-centered curriculum*

In the context of the participants’ experiences, traditional routine-centered program is the challenge of motivating students in the direction of social accountability. Lack of communication upon entering college, curriculum overload, and over-reliance on uncertainties has been part of routine-oriented training. Unfortunately, we have not had much of success due to the practices of some professors and practitioners, such as failure to apply modern approaches to these conditions, the process of transferring knowledge and the practical field. One student talked about the impact of curriculum integration:

*"... An important problem for us is to make connections between different courses or to integrate them for use. Of course, integration can be very good but it's not enough and we do not feel that the subject is being integrated and retained and again it is the same routine old school program and memorizing..."* (Participant No. 12).

Promoting community-based education at university, according to research participants, attention to community-based education at university in recent years at the level of macro and micro programs has enabled us to promote motivation in students to assume future responsibilities, based on their current practical, theoretical and clinical training status in education. Community-oriented education responds to the needs and expectations of society. In other words, to be community oriented is to be literally responsive. Accordingly, any decision made at any level of the education system should contribute to the ability of education to respond to the needs and expectations of the community. In this regard, one of the professors says:

*“.. Being community-based and being accountable is a strategy we must always strive to achieve, and it is important for the student to develop a community-based perspective in order to prepare himself or herself in the future to meet the needs of the people, while the traditional routine of programs cannot help accountability be realized without a real needs assessment...” *(Participant No. 26).

### *Effective evaluation*

 Participants in their experiences have shown that assessment at the two levels of students and professors is performed in two stages in terms of its impact on both student motivation, and on the professor’s effort to empower him / her to respond to students, universities, and society. It is usually possible to gather the necessary information about the professor’s educational evaluation and to compare the obtained information with standard or predetermined criteria, but how this evaluation is performed is crucial to know and students may not have enough benefit from the evaluation results. . One student commented on this and his professor’s feedback:

*"... A good evaluation helps to learn and improve it. It also points us to future responsibility, but it seems some of the evaluations are not useful and are not in line with the responsibilities and goals of medical education, like the feedback from some professors that is not productive at all. ..”* (Participant No. 17).

### *Theme 3: Teaching and learning strategy*

Based on data from participants' experiences on learning and earn practicing competence, they experience a variety of activities such as self-education, formal classroom education, laboratory and patient clinical practice. This important and pivotal class of interaction strategies in the present study paves the way for motivation and accountability among students such that some of them started from self-study and have finally come to take official education seriously. There are some students who regularly switch between the three modes of self-centered learning, participatory learning strategies and instructional strategies through the hidden curriculum, while some others do not achieve sufficient ability and competence to meet the needs of the community in the future. This theme is named observational learning because in all student learning modes they pays more attention to the behavior of the teacher more than their speech, and also in all learning strategies he makes a comparison between himself and others. 

One student participating in the field said:

*"I have experienced and benefited from both individual and participatory learning. But when Dr. ... comes to class and speaks, you feel that he does a lot of effort so that guys can be trained to feel responsible. He likes to be able to convey everything he knows. it is how an experienced and dignify professor is, and we learn from him more because he honestly says I am a responsible physician and this sense of responsibility has caused me to attend this class with all the strength and excitement , and we conclude that he is also responsible and accountable to patients "* (Participant No. 11).

Models, playing both positive and negative roles, have an effect in mastering earning competence. One professor says about the role of models:

*“... Teachers are sometimes role models and mentors for students, and this will have an impact on their future career. For example, Master ... has been to me as a mentor and effective in my work process - how I can put myself in the path that they have gone. This was also helpful. The converse of this is also true. There were also some professors who do not have that good guidance. ... For some reason ... they also help you say I don't want to be like this ...” *(Participant No. 21).

### *Theme 4: Creating purposeful beliefs and behavior*

According to the participants' views and continuous comparison of the data, comparing classes and subcategories with each other, beliefs have a central purpose in the process of motivating students toward social accountability. This pivotal event is due to the context and hegemony of the medical field in society and how the student chooses and selects different strategies, from self-education to formal and participatory strategies. The result of these actions is reactions that show the continuity of accountability under the circumstances, in three dimensions of Respect for social values and norms, Social justice and Serving the benefit of society.

### *Respect for social values and norms*

According to the study participants, accountability beliefs are first created in students, at the beginning of the medical school, and then purposeful behavior is formed based on beliefs following education in classroom and clinical education. According to the participants' ideas and experiences to create accountability behavior in students, the first days of entry into the field should also be spent to treat the student as a responsible person, who is responsible for their own behavior. They decide to attend classes and if they do not attend, and or has an academic failure, they should analyze the situation and seek help. Of course, the university also has some solutions in place, such as an adviser, but it is useful when the student is motivated to seek help. Creating scientific cohesion in the teaching of students occurs in the course of physiopathology, while the student is applying basic knowledge about pathology and diseases. In this stage, professor has a role of a friend, a researcher, an advisor, a friend, and feels like as patron, and after the student enters the clinical course in search of this role, the teachers act as a mirror and reflects the respect for social values and norms in their students. However, unfortunately the professor does not have enough time for this interaction to continue and the student may become frustrated and bored. 

### *Attention to the benefit of patients and society*

students meet a professor in a real sense and in the clinical course, as a role model and leader, and they shape students’ ability, and after reaching the goal, they are gradually back to society to become a capable individual or medical practitioner who is ready to serve and who has a sufficient motivation to respond. In this sense, they pay attention to the benefit of society.

 . One of the students said:

*"... After almost 5 years of looking for a professor to listen to me, I was introduced to Master ... At that time I had no focus at all and didn't know what I was looking for. A professor who has no likes in his ethics and behavior the same as his science; he helped me a lot… he taught me the principles of being a good physician. He was a master for the students, patients and society…he never hesitated and ...”* (Participant No. 21).

Based on the experiences of the professors in this case, it is confirmed that the student becomes responsible when the belief is formed in him achieving competent and clinical governance in healthcare system and drive teachers and students to be competent. 

*"... When I teach a science student about a subject such as diabetes and then teach them the highlights of managing a diabetic patient in all cases and how to communicate and teach this patient, in fact, I awaken the idea of responsibility in students. When I know that all problems relating to patient care must be clear and documented and the community is sensitive to patient rights, I know that I and my students must be held accountable. …” *(Participant No. 24).

## Discussion

Four categories emerged from the results of this qualitative study: social hegemony of medicine, the reality of medical school curriculum, learning strategy and creating purposeful beliefs and behaviors as the core variable of this study. Participants identified that students come into the medical education program motivated by some important factor from the society, which affects their clinical education contents and individual motivation. Individual motivation, self-awareness and helping people were among the medical students’ strong internal motivators. In the context of Iranian social status of medicine, the fact that physicians save lives has a great impact on students' motivation and responsiveness to community needs and different studies confirmed it ( 22 - 26 ).

 In a quantitative focus group study, McCrea et al. aimed to investigate students’ perception of social accountability. The students revealed limited appreciation of the concept of social accountability and acknowledged little explicit teaching underpinning the core concepts such as awareness of local health needs, advocacy, and nurturing of altruism. Nevertheless, the participants considered many aspects of the course and learning initiatives as affecting their attitudes towards this concept implicitly ( 27 ).

In this study, medical students and faculty members also declared the role of professional identity as important in influencing students’ motivation. Students’ personalities, knowledge, skills, creativity, professional communication, and responsibility are largely shaped by their effort to be motivated by team working, awareness of future career and cognitive flexibility, in line with other studies such as Adams et al. ( 28 ).

The reality of medical school curriculum belonged to the other categories commonly articulated by the members of this study. All the participants in their experience, well-defined process motivation, as appeal of the content of medical issues , as well as the status of this field in society as the highest level of dignity that attracts the most talented students. There are numerous studies on the explanations for selecting a medical discipline, where in most studies, social dignity and salary are attractive to those who choose this discipline ( 29 - 31 ).

The study of Gilavand et al. also showed that there was a significant positive correlation between the appropriate job position and income level in selecting a field of study and also there is a positive relationship between external motivation and, social prestige, motivate the students to choose a carrier ( 29 ). In this regard, Kooshki et al. concluded that the factors affecting the choice of this field by medical students are community service and become a lifelong learner ( 30 ).

Our study illustrated that the responsibility of medical faculties in clinical education and basic science in formation of knowledge, skills and ability of medical students are important. Positive role models can motivate them too. In line with this study, others have also shown that medical students’ communication with faculty members and being accepted by them were important factors to establish a sense of responsibility in them, other studies have also demonstrated that students rely on faculties for acceptance. Additionally, faculty’s accountability has a key role in students’ learning and personality development ( 31 - 33 ).

The findings of the research showed that the teaching/ learning strategy can act as organizer in the process of education and, as a result, create accountability in the students. It seems that the implementation of the learning-teaching process through efficient and effective instructors can enable students to make the most of their abilities. Clinical professors also have a tremendous effect on improving the quality of clinical education, and they can make clinical experiences enjoyable for the students; also, competent students have the ability to be responsible more than incompetent students ( 33 ).

Based on this study, traditional routines, inadequate assessment and lack of environmental facilities are among the challenges of the process of motivating students for social accountability and each intervenes in action and engagement of motivation, causing anxiety and inability to respond to the needs of society in the future. Traditional routine means not accepting a change in education and turning curriculum to the time before change. Based on the experience of participants, traditional routine threatens the work of the modernist academics and innovative programs and in some cases completely neutralizes them.

In agreement with the findings, a study found that all components of the curriculum such as need assessment, outcome-based learning and new strategy of teaching and learning as the most important aspects, followed by observational learning and hidden curriculum have impacts on motivation for future accountability in medical students. This is in line with other studies ( 34 , 35 ).

Based on the present study’s findings, “Creating purposeful beliefs and behavior” emerged as the core variable that linked the categories, and was latent in the participants’ statements. The formation of responsive identity is predisposed by the relationship of the students with their professors and professional community of medicine ( 36 ). Cruess et al. stated that professional identity of medical students has a direct relationship with student teaching professionalism, and developing schematic symbolic of these processes ( 37 ).

Dealing with difficult conditions might lead to students’ lack of motivation, or demotivation in attaining competence and social responsibility. As in our study, the participants realized that most of the time humanization thoughts directed them to be responsible in field of medicine.

Law et al. conducted a phenomenological study on the factors guiding the physicians toward lifelong health advocates. The results identified the factors that helped to develop their health advocate identity by exposure to social injustice, upbringing, schooling, and specific formative experiences. They also emphasized the ways they persist in their role as lifelong advocates, continuous learning and improvement, self-reflection and self-reflexivity, collaboration and intrinsic satisfaction in the work ( 38 ).

Based on our findings, which demonstrated the complications of clinical learning conditions, suggestions can be made for future areas of research. In addition to this research, several studies are warranted to detect effective factors in clinical learning motivation. Furthermore, based on the findings of this study, it is recommended that action research should be carried out to reinforce the professional identity of nursing students and nurses, and determine how professional identity relates to students’ motivation in clinical education. 

This study proposes that certain personal competence and efforts of student such as talent in content and process motivation of medicine make the social culture of medicine as the contextual dynamics. It helps to figure out how a medical student selects medicine, and responds carefully to their task, which in turn influences the student’ level of accountability in education, especially clinical education, motivating the students to become socially accountable. 

### *Strengths and limitations of study*

This grounded theory study describes the key dimensions of how medical students are motivated for social accountability in medical school from direct reports of a major prestigious university. A limitations of the study is that it was conducted at Shiraz University of Medical Sciences, which has a history of seventy years and may have different results in other universities.

## Conclusion

The concept of creating purposeful beliefs and behavior is the most important aspect for the development of students’ motivation to social accountability. Subsequently, motivation in medical students to be accountable to community needs is the outcome of dealings with medical school reality. Medical social reality such as, how pay attention to the outcomes in the curriculum and traditional routine-centered curriculum in the context of Social culture of medicine which it is so interesting for student’s family and all of Iranian society. In other words, students impress accountable when they have an acceptable level of motivation at the entrance to the university, in medical school will witness responsible behavior and accountability in all components of educational process. In this context, we expect to facilitate the convergence between the teacher, the student and the student accountability process in the context of clinical governance in healthcare system. On the other hand, we consider that there is a necessity to conduct further qualitative studies on the development of strategies and purposeful beliefs and behavior for social accountability training in medical schools. 
